# HPLC profiling of selected phenolic acids and flavonoids in *Salvia eigii*, *Salvia hierosolymitana* and *Salvia viridis* growing wild in Jordan and their in vitro antioxidant activity

**DOI:** 10.7717/peerj.9769

**Published:** 2020-08-26

**Authors:** Hala I. Al-Jaber, Ashok K. Shakya, Zaha A. Elagbar

**Affiliations:** 1 Department of Medical laboratory Sciences, Faculty of Allied Medical Sciences, Al-Ahliyya Amman University, Amman, Jordan; 2 Department of Chemistry, Faculty of Science, Al-Balqa Applied University, Al-Salt, Jordan; 3 Department of Pharmaceutical Sciences, Faculty of Pharmacy, Al-Ahliyya Amman University, Amman, Jordan; 4 Pharmacological & Diagnostic Research Centre, Faculty of Pharmacy, Al-Ahliyya Amman University, Amman, Jordan

**Keywords:** *Salvia eigii*, *Salvia hierosolymitana*, *Salvia viridis*, LC-ESI-MS/MS, Total phenolic content, Total flavonoid content, Antioxidant activity

## Abstract

**Background:**

*Salvia eigii., Salvia hierosolymitana* and *Salvia viridis* are native to the Mediterranean region, and are used in traditional medicine for the treatment of many ailments. In the current investigation, the methanolic extracts obtained from the air dried aerial parts of *S. eigii*, *S. hierosolymitana* and *S. viridis* from Jordan were screened for their total phenolics content (TPC), total flavonoids content (TFC) and their in vitro antioxidant activity. Additionally, the presence of four bioactive phenolic acids including gallic acid, caffeic acid, rosmarinic acid and salvianolic acid B and other seven flavonoids including luteolin-7-O-glucoside, apigenin, apigenin-7-O-glucoside, rutin, nariginin, hesperidin and quercetin was determined using Liquid chromatography-Electron Spray Ionization-Tandom Mass Spectrometry (LC-ESI-MS/MS).

**Methods:**

Antioxidant activity of the obtained three extracts were examined via the DPPH^•^, ABTS^• +^ radical scavenging methods in addition to Ferrous Ion Chelating (FIC) effect. TFC and TPC of the extracts were measured using the aluminum chloride colorimetric method and the Folin-Ciocalteau method, respectively. The presence and concentration of the selected 11 compounds was further determined through LC-ESI-MS/MS.

**Results:**

The results indicated that three *Salvia* species had high total flavonoids content expressed in mg quercetin/g dry extract (*S. heirosolymitana*: 770.85 ±  5.26; *S. eigii*: 520.60 ±  6.24, *S. viridis*: 311.36 ±  4.41). *S. heirosolymitana* had the highest DPPH^•^ activity (0.184 ±  1.22 × 10^−2^ mg/ml) and FIC effect (0.354 ±  0.018 mg/ml). *S. heirosolymitana* had slightly higher ABTS^• +^ scavenging activity than *S. eigii* (0.176 ±  1.16 × 10^−2^ mg/ml; 0.183 ±  0.031 mg/ml, respectively). All 11 compounds were detected in the extracts of the three *Salvia* species. Luteolin-7-O-glucoside was detected in high concentration levels in the three species (1756.73, 21651.36, and 26125.14 mg/kg dry plant; *S. eigii*, *S. hierosolyimitana* and *S. viridis*, respectively), yet rosmarinic acid had the highest contribution to both *S. hierosolymitana* (27124.93 mg/kg) and *S. eigii* (15783.33 mg/kg). Notably, *S. hierosolymitana* and *S. viridis* contained salvianolic acid B (896.11; 890.9 mg/kg).

**Conclusions:**

The three *Salvia* species exhibited good antioxidant activity, especially *S. heirosolymitana* due to its high TPC, TFC, and the presence of high concentration levels of romarinic acid and other phenolic acids and flavonoids. This is the first phytochemical and antioxidant evaluation of *S. eigii*, *S. hierosolymitana* and *S. viridis* from Jordan. Prior to this investigation, no phytochemical investigation on *S. eigii* was reported.

## Introduction

*Salvia* genus, represented by more than 900 species distributed worldwide (Fu et al., 2013; Al-Eisawi, 1998), is considered as one of the most important genera belonging to the Lamiaceae family. These plants are typically 30–150 cm tall, herbaceous, perennial, rarely biennial or annual with attractive flowers in various colors (Topçu, 2006). Different *Salvia* species are used as spices, flavoring agents and are of economic importance in the field of perfumery and cosmetics (Senatore, Apostolides & Piozzi, 2004; Senatore et al., 2006). Acquiring its name from the Latin word “salvare” in reference to the healing and the curative properties of *Salvia* plants (Gören et al., 2006) numerous *Salvia* species (Table 1) are well recognized in folk medicine for their use in the treatment of more than sixty different ailments ranging from aches to epilepsy, and mainly to treat colds, bronchitis, tuberculosis, hemorrhage and menstrual disorders (Topçu, 2006). Moreover, many *Salvia* species were reported to exhibit antimicrobial, estrogenic, antioxidant, antifungal, antiplasmoidal, anti-inflammatory, antitumor and anticholinesterase properties and are used in the treatment of eczema and psoriasis (Moghaddam et al., 1998). Accordingly, different *Salvia* species were the subject of extensive phytochemical and pharmacognostic research for the isolation, characterization of their secondary metabolites (Al-Qudah et al., 2019; Hasan et al., 2016; Lehbili et al., 2018; Al-Jaber et al., 2012) and evaluation of their pharmacological properties (Abu-Dahab et al., 2014; Al-Qudah et al., 2014; Afifi et al., 2016; Marcinek & Krejpcio, 2017; Khare, Upmanyu & Jha, 2019; Güzela et al., 2019). Table 1 lists some *Salvia* plants and their uses in traditional medicine.

**Table 1:** List of the medicinal and ethno-pharmacological uses of some *Salvia* species.

**Species/ Common name)**	**Part used**	**Recommended uses**	**Method of application**	**Reference**
*S. horminum* L./(Clary)	-Leaves & flowering stems	-Gargle for treating sore gums	-Seeds (2 mm wide & 3 mm long) are socked in water or milk for 1 h.	Ghorbani, Naghibi & Mosaddegh (2006)
	-Seeds (moist)	-Treatment of inflammatory eye diseases and cleansing eyes from dusts and straws. Stomach tonic.	-Moisted seeds directly used for cleaning eyes.
*S. hierosolymitana* Bioss./Jerusalem sage	-Seeds	-Skin cancer	-Seeds/50 grams from the ground seeds mixed with 100 grams lanolin a are applied topically once daily on the tumor area	Jaradat et al. (2016)
*Salvia fruticosa* Mill. (Synonym: *S. triloba*)/Mairameyeh	-Leaves, Aerial parts	- Hair tonic antidandruff, weight loss, enhance memory, colic, abdominal pain, sore gums, nervous system disorders, digestive system problems, liver problems, kidney problems, constipation, drowsiness.	-Decoction, infusion	Abdelhalim et al. (2017) and Noubani, Abu Irmaileh & Afifi (2006)
*Salvia miltiorrhiza*/Danshen	-Roots	-Treatment of malignant tumors, neurological, metabolic disorders, lung diseases, CVDs, inflammatory diseases, gynecological diseases, liver diseases, and renal diseases	-Capsules prepared from the roots	Ren et al. (2019)
*Salvia macrosiphon* Boiss.	-Herb, Seeds	-Respiratory tract ailments, diuretic, carminative, anti-flatulent, otic inflammations, for improving gastrointestinal weakness, treatment of diarrhea and intestinal abrasions, treatment of eye disorders.	-Syrup, decoction	Ghorbani, Naghibi & Mosaddegh (2006) and Hamedi et al. (2016)
	-Leaves	-For the treatment uterine painful conditions, headache and inflammations.
*Salvia officinalis*	-Leaves	-Treatment of inflammation, diarrhea, gastrointestinal pains, diabetes, seizure, ulcers, gout, rheumatism, dizziness, paralysis	Decoction, infusion	Ghorbani & Esmaeilizadeh (2017)

**Figure 1:**
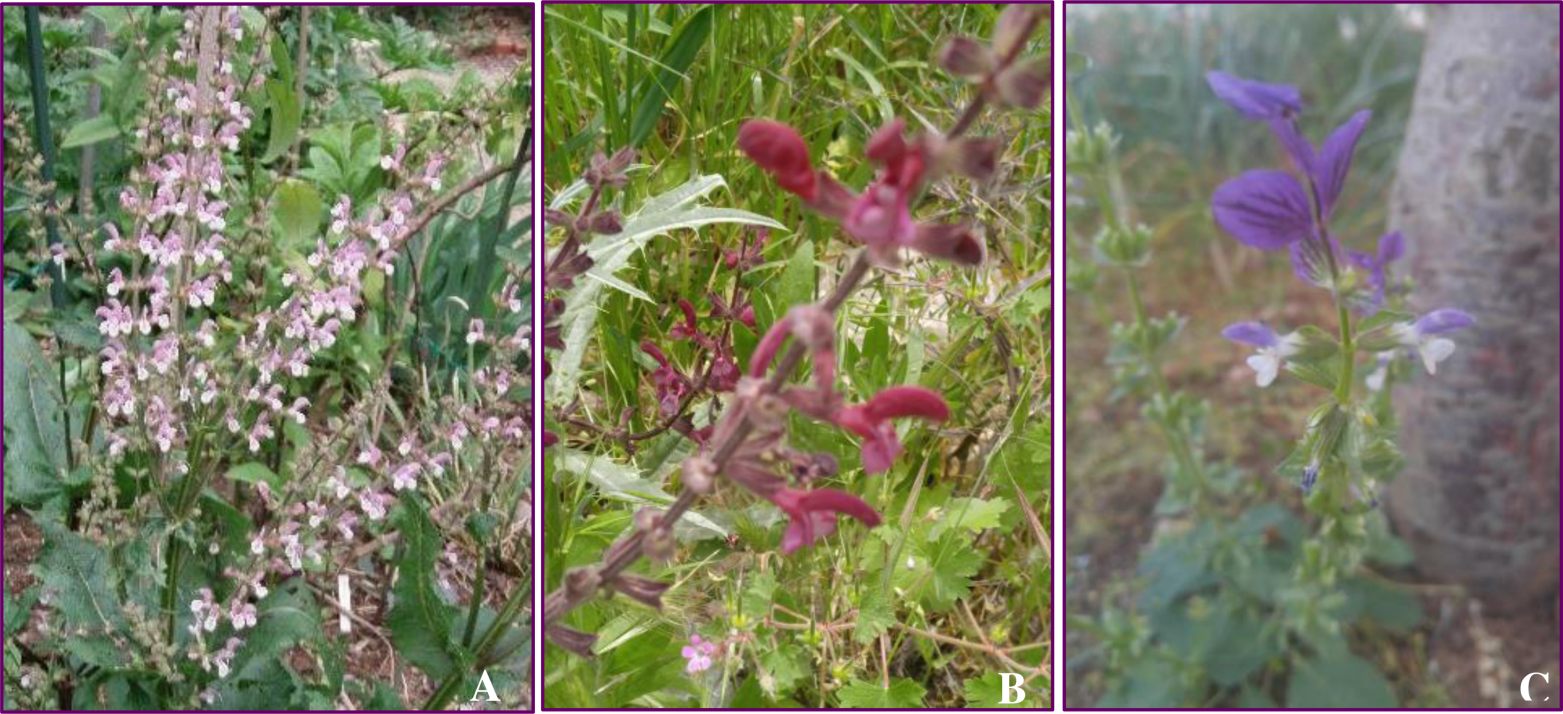
Selected Salvia species in the current investigation, (A) *S. eigii*, source credit: the Victorian Salvia study group; (B) *S. hierosolymitana*; and (C) *S. viridis*.

In Jordan, different *Salvia* species are used in traditional herbal medicine for the treatment of many ailments. Some of these plants are edible while others are used for flavoring tea. There are 25 *Salvia* species reported to grow wild in Jordan including *Salvia eigii* Zohary., *Salvia hierosolymitana* Bioss. and *Salvia viridis* L. (Fig. 1) (Al-Eisawi, 1982).

### *Salvia eigii* Zohry.

*S. eigii* Zohary. is a beautiful showy perennial herb native to the Mediterranean woodlands and shrub lands of Jordan, Palestine, Syria and Lebanon. It grows up to 30–50 cm high and 60–100 cm wide. The flowers have a white lip and the hood is a light mauve to purple. Flowering occurs in spring season during the period extending from March to June. Little literature describes *S. eigii*. The aqueous extract obtained from the aerial parts of this plant has been investigated for Pancreatic triacylglycerol lipase (PL) inhibition and was found to have moderate activity (Kasabri et al., 2014). The extract had also a dual inhibition activity for *α*-amylase and *α*-glucosidase and showed moderate cytotoxic activity against colon cancer cell lines HT29, HCT116 and SW620 (Kasabri et al., 2014). Prior to our study, the plant has never been evaluated for its phytochemical composition and antioxidant activity.

### *Salvia hierosolymitana* Bioss.

*S. hierosolymitanta* Bioss. (known also as the Jerusalem sage) acquired its name from the Greek word “hieros” meaning holy and the Latin name for Jerusalem:“herosolyma”. This plant is native to the East Mediterranean region, growing wild in Jordan, Palestine, Syria, Lebanon, Cyprus and Turkey. It is a perennial herb, 30–60 cm tall, branched from the base with square purple stems. The plant is characterized by purple or red-wine color flowers that are 2–2.5 cm long. Leaves, stems, and floral parts are covered with small hairs. Flowering occurs during the period extending from March to June. The plant is known to grow wild in forest grounds of Irbid, Jerash, Ajloun, Tafila and Amman (Al-Eisawi, 1998). In Levine countries, especially in Jordan and Palestine, *S. hierosolymitana* leaves are edible. Green fresh leaves are boiled, stuffed with rice, minced meat and condiments and then made into rolls, cooked and eaten with yogurt (Al-Eisawi, 1998; Ali-Shtayeh et al., 2008). In Palestinian traditional medicine, grinded *S. hierosolymitana* seeds mixed with lanolin is prescribed for the treatment of skin cancer (Jaradat et al., 2016). Previous phytochemical investigation on the plant led to the isolation of dammarane type triterpenoids (Pedreros et al., 1990), polyhydroxylated triterpenes and rosmarinic acid (De Felice et al., 2006). The methanolic extract obtained from the roots and aerial parts was investigated for its anti-angeogenic activity (Abdallah et al., 2018). The crude ethanolic extract of the plant was evaluated for its cytotoxic activity against MCF-7, T47D, ZR-75-1 and BT 474 cancer cell lines (Abu-Dahab et al., 2012).

### *Salvia viridis* L.

*S. viridis* L. (synonym *S. horminum* L.), commonly known as ‘Red topped sage’, naturally occurs in the Mediterranean region. It is a perennial, annual or biennial herb, having the erect stem of 50 cm and 4–8 axillary flowers (Rungsimakan & Rowan, 2014). *S. viridis* has been used in traditional medicine as gargle against sore gum (Grzegorczyk-Karolak & Kiss, 2018). In Turkey, an infusion of the shoots, flowers, and leaves of *S. viridis* have been used against a sore throat, throat inflammation, antitussive, ulcer, intestinal spasm and gynecological complications (Sharifi-Rad et al., 2018; Zengina et al., 2019).

In continuation of our concern in evaluating the chemical constituents of medicinal plants from the flora of Jordan, we report here the total phenolics and flavonoids content and the antioxidant activity of the methanolic extract obtained from the aerial parts of these three *Salvia* species growing wild in Jordan. Additionally, we also report the quantitative determination of six flavonoids including naringenin, hesperidin, apigenin, luteolin-7-O-glucoside, rutin, quercetin in addition to five other phenolic acids including gallic acid, chlorogenic acid, caffeic acid, rosmarinic acid and salvianolic acid B in the methanolic extract of these three species using Liquid Chromatography-Electron Spray Ionization- Tandom Mass Spectrometry (LC-ESI-MS/MS). Prior to our investigation, no phytochemical studies were carried out on these three species from Jordanian origin.

## Materials & Methods

### Plant material

Aerial parts of each of *S. eigii* Zohary. (Umm Qais–Irbid, 32.6544°N; 35.6881°E), *S. hierosolymitana* Benth. (Ajloun, 32.3327°N; 35.7518°E) and *S. viridis* L. (As Subayhi, Al-Salt, 32.1500°N; 35.7000°E) were collected during their full flowering stage. The plants were identified by Prof. Dr. Hala Al-Jaber, Department of Chemistry, Faculty of Science, Al-Balqa Applied University. A voucher specimen of each species (*S. eigii*: L/SE/2019; *S. hierosolymitana*: L/SHie/2019 and *S. viridis*: L/SH/2019) was deposited at the herbarium of Department of Chemistry, Al-Balqa Applied University, Al-Salt, Jordan.

### Chemicals and standards

All solvents (HPLC grade) used in this investigation, rutin hydrate (≥94% HPLC), nariginin, hesperidin, gallic acid, chlorogenic acid and caffeic acid (HPLC, ≥98%) were obtained from Sigma-Aldrich (Buchs, Switzerland). Luteolin-7-O-glucoside (HPLC, ≥98%), apigenin (HPLC, ≥99%) were products of EXTRASYNTHESE (France). Apigenin-7-O-glucoside, rosmarinic acid and salvianolic acid B were purchased from EDQM (Strasbourg, France).

### Extraction and sample preparation

Extraction of the selected *Salvia* species was performed according to the procedure described in the literature but with slight modification (Fotovvat, Radjabian & Saboora, 2019). Briefly, 10.0 g sample of the air dried and pulverized aerial parts of each selected species was soaked in HPLC-grade methanol (3 × 100 ml, 24 h each) at room temperature. After filtration, the solvents were evaporated under vacuum at 40 °C. The obtained crude extracts were then stored in dark at 4 °C until analysis. For LC-ESI-MS/MS analysis, the dried extracts were dissolved in 70% aqueous HPLC-grade ethanol, filtered and then prepared for analysis. Stock solutions of each extract (4,000 ppm) were prepared and then assayed immediately for their flavonoids and phenolic acid compounds using LC-MS/MS.

### Total flavonoids content (TFC) and Total phenolics content (TPC)

The TFC of the methanolic extracts was determined colorimetrically according to the method described in the literature with slight modifications (Olajire & Azeez, 2011; Al-Jaber, 2017). Briefly, 1.0 mL sample of each extract (1 mg/ml) diluted with 4.0 ml distilled water were introduced into 10 ml volumetric flask and then 0.30 ml of NaNO_2_ solution was added. After 5 min, 0.30 ml of AlCl_3_ solution (10% w/v) was added to the mixture. The solution was incubated 6 min, and then 2.0 ml of 1.0 M NaOH solution was introduced and the final volume of the solution was adjusted to 10.0 ml with distilled water. After another 15 min, the absorbance of the resulting solution was measured at 510 nm using methanol as a blank. The TFC content in the plant extracts was determined and expressed in mg quercetin/g dry extract.

The TPC was determined using the Folin–Ciocalteu method described in the literature (Al-Jaber, 2017). Briefly, to 0.5 ml of extract, 2.5 ml of Folin–Ciocalteu reagent (2N diluted ten folds) and 2 ml of Na_2_CO_3_ solution (75 g/l) were added. After allowing 15 min period of incubation at room temperature, the absorbance of the resulting solution was measured at 765 nm. Methanol was used as a blank reference. The TPC of the different extracts is reported as mg/g gallic acid equivalent. All measurements were performed in triplicates.

### Antioxidant activity

### DPPH^•^ free radical scavenging activity

The antioxidant activity of the methanolic extracts obtained from the three *Salvia* species was evaluated using the DPPH^•^ radical scavenging method according to the procedure mentioned in the literature using ascorbic acid and *α*-tocopherol as positive controls (Olajire & Azeez, 2011; Al-Jaber, 2016; Al-Jaber, 2017). Briefly, 1 ml of 0.1 mM DPPH^•^ solution (dissolved in MeOH) was added to 2.0 ml of each methanolic extract solution at various concentration levels (0.005–0.5 mg/ml). The solutions were incubated in dark for 30 min at room temperature and then the absorbance of the solutions was measured at 517 nm against methanol blank sample. The ability to scavenge the DPPH^•^ radical was calculated using the following equation:

\begin{eqnarray*}{\text{DPPH}}^{\bullet } \text{scavenging effect}  \left( \text{%} \right) = \frac{Ac-As}{Ac} \times 100\text{%}. \end{eqnarray*}

Where *A*_*C*_ is the absorbance of the blank and *A*_*S*_ is the absorbance in the presence of the extract/compound. The scavenging activity assay of all extracts, positive controls and IC_50_ value determinations were conducted in triplicates using the non-linear regression analysis of the GraphPad Prism 6 (GraphPad Software, San Diego, CA, USA). The UV- spectra were recorded on Wavelight II UV- visible spectrophotometer (Version 7120V1.8.0, USA).

### ABTS radical scavenging assay

The total antioxidant activity determined by radical cation (ABTS^• +^) decolonization assay was performed according to the procedure described in the literature with slight modifications (Zhao et al., 2011; Olajire & Azeez, 2011). The radical cation reagent (ABTS^• +^) solution was prepared by mixing similar quantities of 7 mM of ABTS^• +^ and 2.4 mM of potassium persulfate (K_2_S_2_O_8_) solutions and allowing them to react in dark for 16 h and at room temperature. Before use; this solution was diluted with methanol to get an absorbance of 0.75 ± 0.02 at 734 nm. To measure the antioxidant power of the plants’ extracts, 3 ml of ABTS^• +^ reagent solution and 1 mL of each extract at various concentration levels (ranging from 0.005–0.500 mg/ml) were mixed and the absorbance was then measured at 734 nm on Wavelight II UV- visible spectrophotometer (Version 7120V1.8.0, USA). Blank sample was run in each assay and all measurements were done after at least 5 min. Ascorbic acid and *α*-tocopherol were used as positive controls. All measurements and IC_50_ value determinations were conducted in triplicates.

### Ferrous Ion Chelating (FIC) effect

The ability of the tested extracts and EDTA used as a positive control to chelate ferrous ion from the formation of ferrozine-Fe^2+^ complex was determined according to the procedure listed in the literature (Al-Qudah et al., 2019). Briefly, a 3 ml of methanol solution containing different concentrations of the extracts (0.005–1.0 mg/ml) was added to a 0.25 ml of 2 mM ferrous chloride (FeCl_2_) reagent. Then, 0.2 ml of 5 mM ferrozine solution was added to the mixture and after vigorous shaking, the mixture was allowed to stand at room temperature for 10 min. The reduction in the absorbance of the red colored complex was measured at 562 nm. All measurements and IC_50_ values determination for the tested extracts and the positive control were conducted in triplicates.

### LC-MS/MS analysis parameters

Analysis of flavonoids and phenolics compounds was performed on HPLC (Agilent 1200 series, USA) equipped with LC-ESI-MS/MS API 5000 system (AB Sciex Instrument, Framingham, USA) utilizing Analyst 1.6.3 software for data analysis. The chromatographic separation was conducted at 40 ± 1 °C using Inertsil ODS-3 column (4.6 × 150 mm, 5 µm, GL Sciences-Japan) at ambient temperature. The elution gradient consisted of mobile phase A (5 nM ammonium formate in water: Methanol (90:10; v/v)) and solvent B (5 mM ammonium formate in methanol). The gradient program with the following proportions of solvent B was applied (%B, min): 40–90% B (0.00–6.00 min), isocratic 90% (6.00–10.00 min), isocratic 40% (10.01–15.00 min). The solvent flow rate was 0.5 ml/min and the injection volume was 10 µl. MS/MS analysis was performed in negative ion mode with an ion spray voltage of −4,500 V. Nitrogen gas at a pressure of 60 psi was used as the nebulizing and drying gas. The mass spectra were obtained over the *m/z* range of 100–1,000 amu.

### Preparation of standard solution

A stock solution containing eleven standard compounds (0.5 mg/ml) was prepared in HPLC-grade methanol and diluted to eight different concentrations to construct the calibration curve.

## Results

### Extraction yield, TPC, TFC and antioxidant activity

The results of extraction yields, TPC and TFC and the antioxidant activities of the methanolic extracts of the three *Salvia* species are shown in Table 2.

**Table 2:** Extraction yield, total phenolic content (mg gallic acid/g dry extract), total flavonoids (mg quercetin/g dry extract), and Antioxidant activities using DPPH, ABTS radical scavenging, and ferrous ion (FIC) chelating assay methods. Values expressed are means ± SD of three parallel measurements.

**Plant**	**Yield (g/gDW)**^a^	**TPC**	**TFC**	IC_50_ (mg/mL)		
				**DPPH**	**ABTS**	**FIC**
*S. eigii*	9.00	24.99 ± 3.12	520.60 ± 6.24	0.248 ± 1.28 × 10^−3^	0.183 ± 0.031	0.433 ± 0.055
*S. hierosolymitana*	1.033	27.31 ± 0.92	770.85 ± 5.26	0.184 ± 1.22 × 10^−2^	0.176 ± 1.16 × 10^−2^	0.354 ± 0.018
*S. viridis*	6.40	18.51 ± 0.32	311.36 ± 4.41	0.234 ± 4.93 × 10^−3^	0.203 ± 5.02 × 10^−3^	0.476 ± 0.070
*α*-Tocopherol	–	–	–	3.96 × 10^−2^± 1.0 × 10^−3^	4.22 × 10^−2^± 1.00 × 10^−3^	–
Ascorbic acid	–	–	–	1.20 × 10^−3^± 1.3 × 10^−4^	2.66 × 10^−2^± 7.3 × 10^−4^	0.501 ×±0.098
EDTA	–	–	–	–	-	1.14 × 10^−3^± 2.0 × 10^−4^

**Notes.**

a DW, dry weight plant material.

The results in the table showed that *S. heirosolymitana* had the highest TPC (27.31 ± 0.92 mg gallic acid /g dry extract), TFC (770.85 ± 5.26 mg quercetin/g dry extract) followed by *S. eigii* (24.99 ± 3.12 mg gallic acid/g dry extract; 520.60 ± 6.24 mg quercetin/g dry extract). The obtained results clearly indicated that *S. hierosolymitana* had the highest radical DPPH^•^ scavenging activity.

### LC-MS/MS profiling of phenolic acids and flavonoids

The results of the LC-MS/MS analysis of the three species are summarized in Table 3. LC-MS/MS analysis of the methanolic extract of the three *Salvia* species revealed that the three *Salvia* species were rich in caffeic acid derivatives, mainly rosmarinic acid. Among the different compounds studied, the plants also contained high concentration levels of luteolin-7-O-glucoside (Table 3).

**Table 3:** LC-MS/MS data for standard phenolic and flavonoids compounds detected in *S. eigii*, *S. hierosolymitana* and *S. viridis* from Jordan and their concentration (mg/kg dry plant material).

**Compound**	**Structure**	**Formula**	***R*_*t*_ (min)**	**MS/MS**	**Concentration (mg/kg dry plant material)**		
					***S. eigii***	***S. hierosolymitana***	***S. viridis***
Gallic acid	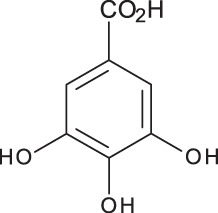	C_7_H_6_O_5_	3.46	169, 125	100.09	84.15	27.36
Salvianolic acid B	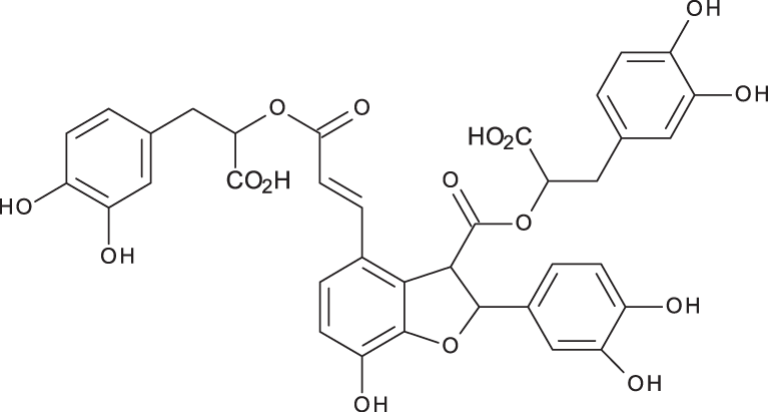	C_36_H_30_O_16_	3.51	717, 519	13.79	896.11	890.90
Chlorogenic acid	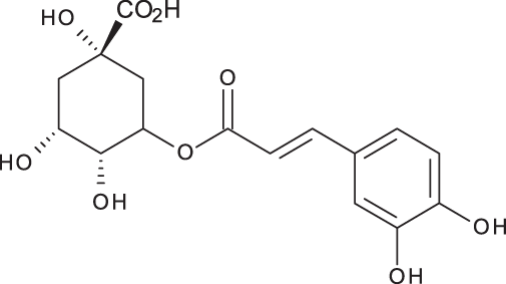	C_16_H_18_O_9_	3.80	353, 191	173.75	201.19	6619.93
Caffeic acid	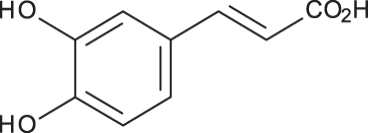	C_9_H_8_O_4_	4.15	179, 135	32.97	260.79	453.03
Rosmarinic acid	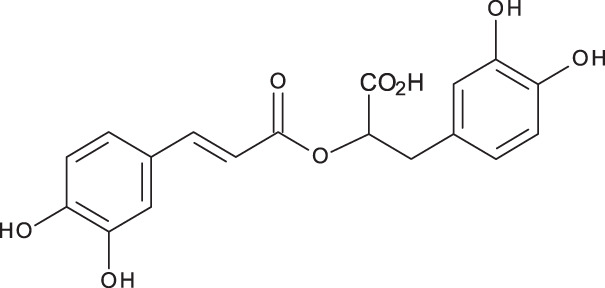	C_18_H_16_O_8_	5.00	359, 161	15783.33	27124.93	329.91
luteolin-7-O-glucoside	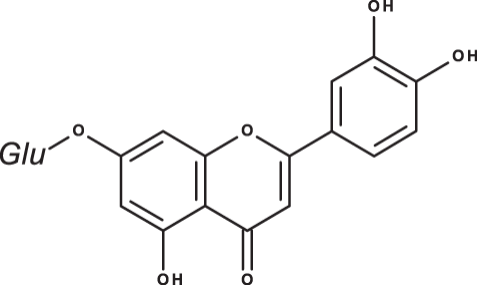	C_21_H_20_O_11_	7.45	447, 285	1756.73	21651.36	26125.14
Hesperidin	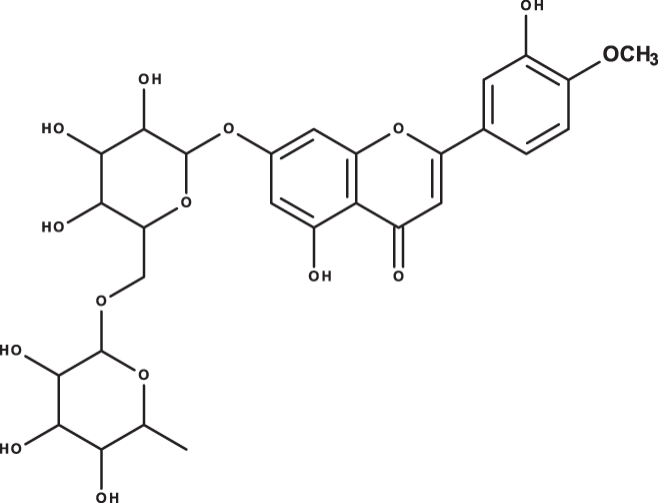	C_28_H_34_O_15_	7.58	609, 300	142.74	95.69	298.38
Rutin	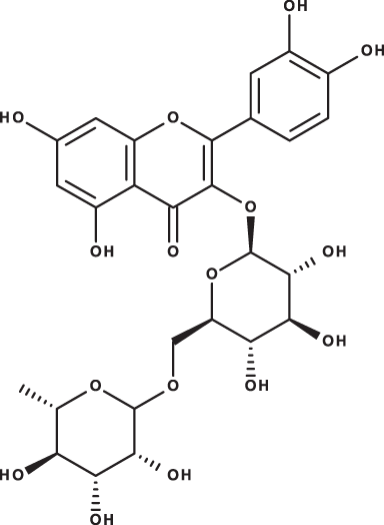	C_27_H_30_O_16_	7.59	609, 300	54.49	3.71	100.89
Quercetin	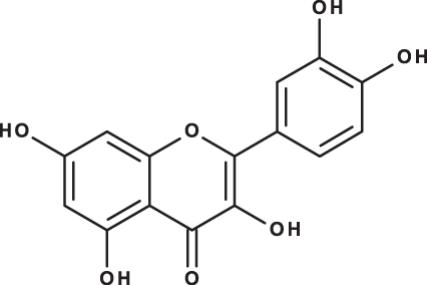	C_15_H_10_O_7_	9.56	301, 179	6.10	6.91	7.10
Apigenin	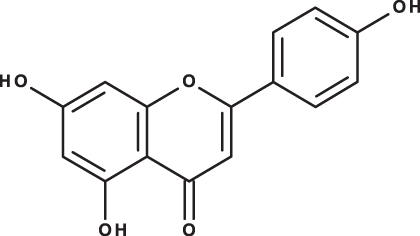	C_15_H_10_O_5_	10.53	269, 117	111.31	26.08	39.37
Naringenin	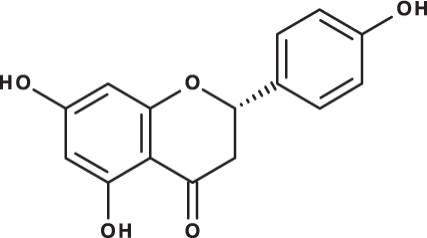	C_15_H_12_O_5_	10.55	271, 150	154.02	418.59	67.77
				**Total**	**18329.32**	**50769.51**	**34629.87**

As could be deduced from the LC-MS/MS results (Table 3), all eleven compounds were detected in the three species, but with variable concentration levels. Careful inspection of the results indicated that the most abundant component detected in *S. hierosolymitana* and *S. eigii* was rosmarinic acid (27124.93, 15783.33 mg/kgDW, respectively). *S. heirosolymitana* also contained high concentration levels of luteolin-7-O-glucoside (21651.36 mg/kgDW), naringenin (418.59 mg/kgDW), caffeic acid (260.79 mg/kgDW) and had the highest content of salvianolic acid B (896.11 mg/kgDW) when compared to the other two species. *S. eigii* was richer in each of gallic acid (100.09 mg/kgDW) and apigenin (111.31 mg/kgDW) and contained luteolin-7-O-glucoside detected at lower concentration level compared to *S. hierosolymitana* (1756.73 mg/kgDW). On the other hand, *S. viridis* had the highest content of luteolin-7-O-glucoside (26125.14 mg/kgDW), chlorogenic acid (6619.93 mg/kgDW), caffeic acid (453.03 mg/kgDW), hesperidin (298.38 mg/kgDW) and rutin (100.89 mg/kgDW).

## Discussion

In the current investigation, a methanol extract was prepared from the aerial parts of the three *Salvia* species and each extract was assayed for its TPC, TFC and its antioxidant activity using DPPH^•^, ABTS^• +^ and FIC methods. Results obtained (Table 2) indicates that the plants showed high TFC especially for *S. hierosolymitana* (770.85 ± 5.26 mg quercetin/g dry extract). The methanolic extract of *S. hierosolymitana* had also had the highest TPC (27.31 ± 0.92 mg gallic acid/g dry extract) compared to the other two species.

Many assay methods are described in the literature for the in vitro evaluation of the antioxidant activity of plants crude extracts (Leong & Shui, 2002; Jeong et al., 2012) that are basically classified according to the mechanism of action by which antioxidant active compounds stop the chain-breaking reactions. Based on this classification, two main groups are identified including the Hydrogen-Atom Transfer Reactions (HATR) and the single electron transfer reactions (SETR) (Chaves, Santiago & Carlos Alías, 2020). In the current investigation, the antioxidant activity of the methanolic extract of the three *Salvia* species was investigated using the DPPH^•^, ABTS^•^ and FIC assay methods, all belonging to the SETR group. The obtained results (Table 2) clearly indicated that *S. hierosolymitana* had the highest antioxidant activity in all assay methods due to its high TFC and TPC, especially caffeic acid derivatives as could be concluded from LC-MS/MS results (Table 3).

*Salvia* species are known to be rich in phenolic acids and flavonoids. Different phytochemical investigations showed that the aerial parts including leaves and flowers are mainly rich in flavonoids, tritpernoids and monoterpenes while the roots are rich in diterpenoids.

Rosmarinic acid has been detected in many *Salvia* species at various concentration levels. It was detected in the range 5.5–39.3 mg/g dry weight in *S. officinalis* from China, its content varied depending on the site of collection and extraction method (Wang, Provan & Helliwell, 2004; Bandoniene, Murkovic & Venskutonis, 2005; Grzegorczyk, Matkowski & Wysokińska, 2007; Shekarchi et al., 2012; Dent et al., 2017). Rosmarinic acid content has also been detected lately in 14 *Salvia* species growing wild in Anatolia-Turkey (1.08–18.7 mg/g dry weight) and the highest content was reported in *S. caespitosa* (Adımcılar et al., 2019). In Iran, Fotovvat, Radjabian & Saboora (2019) investigated the content of some selected phenolic compounds in 41 different *Salvia* populations. In this study, rosmarinic acid has been detected at lower concentration levels in the roots (0.62 ± 0.13–11.56 ± 0.35 mg/gDW) when compared to leaves (0.45 ± 0.03–41.53 ± 0.88 mg/gDW), highest content occurred *S. verticillata*. This compound was also detected in the leaves of *S. glutinosa* from Lithuania (47.3 mg/g dry weight) (Bandoniene, Murkovic & Venskutonis, 2005). Lately, it was detected in the aqueous extracts obtained from the aerial parts of each of *S. Africana*, *S. officinalis* ‘Icterina’ and *S. mexicana* from Portugal (77.0 ± 3.6, 52.7 ± 0.5, 29.4 ± 0.6 mg/gDW) (Afonso et al., 2019). The content of rosmarinic acid has been reported in *S. viridis* from various origins. It was detected at low concentration levels in the methanolic extract obtained from *S. viridis* leaves from Iran (1.15 ± 0.61 mg/g dry weight) and was not detected at all in the roots methanolic extract (Fotovvat, Radjabian & Saboora, 2019). Grzegorczyk-Karolak & Kiss (2018) determined the content of this compound in the hydro-ethanolic extract obtained from *S. viridis* shoots (1.267 ± 0.058 mg/g dry weight aerial parts) and herbal preparations including infusion (1.283 ± 0.050 mg/g dry weight aerial parts) and decoction (0.525 ± 0.145 mg/gDW).

The isolation and characterization of caffeic acid from *S. viridis* L. cvar. Blue Jeans from Thiland has been reported (Rungsimakan & Rowan, 2014). Caffeic acid has been detected in the methanolic extract obtained from the leaves of *S. viridis* from Iran (3.20 ± 0.18 mg/g dry weight) (Fotovvat, Radjabian & Saboora, 2019). Recently, Zengina et al. (2019) investigated the effect of different extraction methods on the phenolic acids and flavonoids profiles of the roots extract of *S. viridis* from Turkey (Zengina et al., 2019). The results reported qualitatively the detection of gallic acid, caffeic acid, luteolin-7-O-glucoside, rosmarinic acid, naringenin in the ethanolic ultrasonic assisted root extract of *S. viridis* compared to other extraction methods.

## Conclusions

Prior to our investigation, no phytochemical investigation for *S. eigii* was reported. Moreover, this is the first quantitative determination of the described eleven compounds in these three species from Jordanian origin.

Among the different phenolic acids and flavonoids determined in this investigation, the present study confirmed that the aerial parts of *S. eigii*, *S. hierosolymitana* and *S. viridis* from Jordan, are highly valuable sources for rosmarinic acid and luteolin-7-O-glucoside. In the current investigation, the methanolic extract of *S. heirosolymitana* had relatively the highest DPPH^•^ and ABTS^• +^ radical scavenging power and showed also high FIC effects as compared to the methanolic extracts of both *S. eigii* and *S. viridis* from Jordan. The observed high antioxidant activity observed for this plant was mainly attributed to the presence of high concentration levels of rosmarinic acid, caffeic acid, salvianolic acid B, luteolin-7-O-glucoside and naringenin.

##  Supplemental Information

10.7717/peerj.9769/supp-1 Supplemental Information 1 Raw data for antioxidant activity

10.7717/peerj.9769/supp-2 Supplemental Information 2 Raw data for TPC and TFC of the three plants

10.7717/peerj.9769/supp-3 Supplemental Information 3 Chromatograms of standard compounds and plant species

## Additional Information and Declarations

### Data Availability

The following information was supplied regarding data availability: Raw data for TPC, TFC, antioxidant activity and chromatograms of standards and plant extracts are available as Supplemental Files.
